# Implementation of a Case Presentation Program for Clinical Nutrition Students

**DOI:** 10.3389/fnut.2022.845030

**Published:** 2022-04-11

**Authors:** Shaahin Shahbazi, Maryam Vahdat Shariatpanahi, Saba Vahdatshariatpanahi, Erfan Shahbazi, Zahra Vahdat Shariatpanahi

**Affiliations:** ^1^Department of Gastroenterology, Faculty of Medicine, Ilam University of Medical Sciences, Ilam, Iran; ^2^Department of Psychiatry, Faculty of Medicine, Azad University of Medical Sciences, Tehran, Iran; ^3^Simon Fraser University, Burnaby, BC, Canada; ^4^School of Medicine, Tehran University of Medical Sciences, Tehran, Iran; ^5^Department of Clinical Nutrition and Dietetics, Faculty of Nutrition and Food Technology, National Nutrition and Food Technology Research Institute, Shahid Beheshti University of Medical Sciences, Tehran, Iran

**Keywords:** case-based learning, questionnaire, student, clinical nutrition, consultation

## Abstract

**Background and Aims:**

To implement a training method increase clinical nutritionists' knowledge and make doctors more familiar with this specialty.

**Methods:**

The study was conducted in an internship course of all third semester clinical nutrition students. At first, conventional training was presented for students, and then, in the same duration, case presentation training program was implemented. The presentations were filmed and uploaded to the Internet, and the link was placed on the hospital's website. At the end of the 2 phases, the students were asked to answer the survey questionnaire. Additionally, consultation report sheets were evaluated and scored by 2 physicians. Number of consultation requests was also recorded in 2 study phases.

**Results:**

The mean satisfaction score was statistically higher in the case presentation training program than in the conventional program. All the students recommended similar case report program courses for the students in the future. Although the mean consultation report score was not statistically different between the two training programs, case presentation program resulted in significantly better scores in 4 items of nutrition focused physical examinations, assessment of malnutrition, assessment of related laboratory tests, and food-drug interactions. Number of consultation requests was significantly increased during the case presentation program training compared to the conventional training from 194 to 272 (*P* < 0.001).

**Conclusion:**

From the students' perspective, the case-based learning report was preferred to the conventional method. From the physicians' viewpoint, the answer to the counseling sheets was more complete and helpful.

## Introduction

Clinical nutrition as part of the process of disease management has become highly important (1). In fact, the clinical nutritionist is a member of the treatment team. Each hospitalized patient needs nutritional care. Ideally, a team of doctors, nurses, nutritionists and pharmacists should work together to provide patients with care (2). When the staff work as a team, it is more cost-effective, more efficient, and ultimately with reduced mortality and morbidity (2). The importance of malnutrition in suppressing the immune function and disease progression is well-known (3). An important duty of clinical nutritionists is to diagnose patients with malnutrition at admission in the hospital and the occurrence of malnutrition during hospitalization (4). Performing this task requires full knowledge of the patient. This knowledge includes taking a complete medical and food intake history, nutrition focused physical examination, anthropometric measurements, interpretation of related laboratory tests, and assessment of food and drug interactions (5). Furthermore, the significance of clinical nutrition for many physicians is still unclear (6).

The Master Program of Clinical Nutrition, which has been taught for more than 50 years in many countries, has been launched in our country since 2017.

In our hospitals, unfortunately, nutrition counseling is provided at a low level, and minor importance is attached to clinical nutrition. Therefore, we aimed to implement a training method to increase clinical nutritionists' knowledge and make doctors more familiar with this specialty. This program was a case report presentation, filming it and uploading it to the hospital's educational website to be seen by physicians, nutrition students, and other students.

This study aimed to present a teaching program to obtain a complete and uniform history and physical examination, to increase the students' ability to gain and analyze information about the patients, and to familiar the physicians with the key role of clinical nutritionists.

## Materials and Methods

### Participants and Intervention

The study was conducted from 23 September 2019 to 20 February 2019 on all third-semester students of clinical nutrition at Taleghani University Hospital. They were eight female students entering the hospital to undergo clinical courses. Annually, 8 clinical nutrition students in the third semester are admitted to our university to spend their internship course. These students receive their theoretical training in the two first trimesters. Initially, we intended to conduct this study in two different entrances in a duration of 3 semesters, but due to the incidence of COVID-19 disease, this study was conducted only on one group of students in 2019. The third semester of clinical nutrition students, which is their internship phase, was divided into three phases. In the first phase, with a duration of 10 days, the necessary training was provided, including familiarity with the medical record, how to extract important points from the medical record, how to communicate with the staff, how to communicate with the patient, how to take a history, diet history, physical examination of the patient, how to interpret the related laboratory data, food-drug interaction, and how to respond to physicians' consultation. The second and third phases were the studied phases with equal duration, in which the counseling was answered by the students themselves.

In the second phase, training was provided traditionally. Patients who had nutrition counseling, were visited by the professor (clinical nutritionist) and students, and necessary training and guidance on response to consultation were provided.

In the third phase, all students were instructed how to prepare a case and present it. Each student was assigned 2–3 sessions to present one patient per session. This patient was selected by the mentioned professor. Extraction of information from the medical record, examination of the patient, and preparation of a PowerPoint were carried out with the professor's the guidance. PowerPoint preparation and discussion with the professor took about 3 days. During this time, the students had to perform literature searches to better understand the patients' conditions. She also interacted regularly with the professor. One day before the presentation, the PowerPoint file was reviewed and corrected by the professor. The presentation time was ~20 min, and free time was given to questions asked by the presenter and the professor. The PowerPoint presentation session was filmed with a mobile camera. The professor added English subtitles to the films. Finally, the films were uploaded to the Internet (https://www.aparat.com/nutritiondata). The link to the films was placed on the hospital's website.

At the end of the second and third phases, the students were asked to answer the survey questionnaire. The questionnaire included 9 graded questions using a 5-point Likert scale with the options: strongly agree, agree, neutral, disagree, and strongly disagree (score 5 to score 1). Table 1 shows the questionnaire presented to the students.

**Table 1:** Survey questionnaire given to clinical nutrition students before and after the intervention.

	**Conventional training**	**Case report program**	***P*-value**
The course was well organized.	4.12 ± 0.35	5	<0.001
I encouraged and provided opportunities for discussion.	4.62 ± 0.51	5	0.08
The course improved my understanding.	4.12 ± 0.99	5	0.04
The course helped me in searching the literature and identifying relevant learning resources.	3.75 ± 0.16	5	<0.001
The course helped me in recognizing my strengths and weaknesses.	4.5 ± 0.75	5	0.10
The course improved my presentation skills.	3.37 ± 0.51	5	<0.001
The course boosted my confidence.	3.50 ± 0.53	5	<0.001
The course made me feel more prepared for work in the hospital independently.	3.62 ± 0.91	5	0.004
I recommend this course for the students in the future.	2.87 ± 0.64	5	<0.001
Total score	3.83 ± 0.59	5	<0.001

### Consultation Report Sheet

Consultation report sheet had 14 components, including: date and time of the consultation; history; nutrition focused physical examinations; related anthropometric measurements; assessment of malnutrition; calculation of calorie requirement; recording macronutrients requirements; recording micronutrients requirements; assessment of related laboratory tests; food and drug interaction; listing the result of consultation; name of the responsible faculty member; stamp, signature, and attachment of the diet sheet. Consultation report sheets were scored using a 3-point Likert scale with the options: complete (score of 1), incomplete (score of 2) and poor (score of 3) with scores ranging from 0 to 42. Two university physicians scored the consultation report sheets in a blinded way, and their mean scores were recorded as shown in Table 2.

**Table 2:** Scoring consultation reports before and after the intervention.

	**Conventional training**	**Case report program**	***P*-value**
Date and time of the consultation	3	3	1
History	2.25 ± 0.88	2.74 ± 0.46	0.27
Nutrition focused physical examinations	2.12 ± 0.99	2.87 ± 0.35	0.04
Related anthropometric measurements	3	3	1
Assessment of malnutrition	1.75 ± 0.70	2.62 ± 0.51	0.04
Calculation of calorie requirement	3	3	1
Calculation of macronutrients	3	3	1
Calculation of micronutrients	3	3	1
Assessment of related laboratory tests	2 ± 1.06	2.75 ± 0.46	0.04
Food and drug interactions	1.87 ± 0.83	2.75 ± 0.46	0.04
The result of the consultation is listed	3	3	1
The name of the responsible faculty member	3	3	1
Stamp and signature	3	3	1
Attachment of diet sheet	2.50 ± 0.75	2.62 ± 0.51	0.35
Total score	2.60 ± 0.86	2.88 ± 0.45	0.42

### Outcomes

The outcome measures were: (1) Investigation of the students' perceptions of learning a case report program using a five-point scale; (2) Assessment of the students' performance by evaluating the consultation report sheets; (3) Feedback of the hospital physicians by number of consultation requests.

### Statistical Analysis

The SPSS version 21 was used for statistical analyses. *P* < 0.05 was considered significant. Kolmogorov-Smirnov test showed the normal distribution of data, and the paired-sample *t*-test was used to determine the difference between items of two methods of training and consultation reports.

The reliability of the questionnaire was determined using the Cronbach alpha coefficient by 8 senior clinical nutrition students, which was higher than 0.8.

To assess the qualitative face validity, the questionnaire was evaluated by 8 senior clinical nutrition students. They examined the questionnaire in terms of relevancy, ambiguity, and difficulty. For qualitative content validity, the questionnaire was distributed among 10 experts composed of nutrition professors and statistics specialists. The changes were made in the questionnaire according to their opinions about the appearance, grammar, wording, item allocation, scaling, writing style of questions, and putting the proper words in the sentence.

## Results

All the eight students were female. As Table 1 shows, the mean scores of 7 items out of 9 items of the survey questionnaire were significantly higher in the case report program than in the conventional program. All students recommended similar case report program courses for the students in the future. All of them strongly agreed that the course boosted their confidence and improved their presentation skills. All of them agreed that the course improved their literature searching skills and identification of relevant learning resources. All the students claimed that this training had prepared them to enter the employment.

Table 2 presents the results of consultation reports. Although the average consultation report scores were not statistically different between the two training programs, the case report program training resulted in significantly better scores for 4 items of consultation reports. These items were nutrition focused physical examinations, assessment of malnutrition, assessment of related laboratory tests, and food-drug interactions.

We also compared the rate of nutritional consultation request during the second and third phases of the study periods. The results indicated that number of consultation requests was significantly increased during the case report program training compared to the conventional training from 194 to 272. In other words, it was about 24 consultation requests vs. 34 consultation requests for each student in the second and third phases sequentially (*P* < 0.001).

## Discussion

The present study demonstrated students' high degree of satisfaction with the case report program training in improving their skills, knowledge, self-esteem, and preparation for work independently in the hospital. Moreover, all of them recommended this course for the students in the future. In addition, the quality of response to consultations was increased. Furthermore, the physicians' feedback assessed by number of consultations was excellent owing to their familiarity with clinical nutrition.

In fact, we attempted to teach clinical nutrition students a complete and uniform model to receive history and physical examination, to interpret with laboratory data, to consider the food-drug interaction, and finally to provide complete results as recommended (7). During this time, the students had to perform literature searches, study the patient, and since their presentations were uploaded to the hospital website, they did their best to ensure that the presentation was complete, up-to-date, and perfect. The students also had to interact more with the professor, so that they used the professor's knowledge and experiences appropriately.

Additionally, the films uploaded to the website made it usable for every clinical nutrition students in other centers whenever they needed them. By viewing the films, the physicians became more familiar with clinical nutrition and the importance of nutrition therapy, as demonstrated by our results.

To the best of our knowledge, no study similar to ours has been conducted on clinical nutrition students; however, some studies evaluated the case-based learning method. In one study conducted on 29 senior students in the clinical nutrition and dietetics course at the University of Sharjah, a case-based learning method of teaching was studied by evaluating students through a pre-test-post-test mechanism. Their results exhibited learning improvements after holding the case-based learning sessions (8). Case-based learning method is more studied and employed in other medical sciences disciplines. The most represented fields are medicine (9), nursing, occupational therapy, allied health, child development, and dentistry (10–12).

Oral presentation is an essential skill for clinical nutritionists (13), and despite its obvious significance, it has not been consistently and effectively taught for clinical nutritionists. In fact, it has received less attention in the educational curriculum. As this method of learning is included in medical education curricula (14), it appears that this method of learning should be strongly considered in clinical nutrition education curricula.

The strengths of our study were that, firstly, this educational method was implemented for the first time, and secondly, with this method, we identified the weaknesses of clinical students and improved their learning. Finally, we were able to draw the attention of other fields, particularly physicians, to this field to emphasize its importance in the medical management of patients. Our most important limitation in this study was the emergence of COVID-19 disease. The study was designed to run in two semesters, but dedicating some parts of the hospital to hospitalizing COVID-19 patients, the sudden reduction in nutrition counseling, and the reduced number of students in the hospital caused us to finish the study quickly. Another limitation of the study was that it was time-consuming for the professor; therefore, the presentation of such teaching methods depends on the professor's motivation. It can be made easier with the help of senior students and their participation in this educational program under the professor's supervision.

## Conclusion

From the students' perspective, the case-based learning report was preferred to the conventional method. From the physicians' viewpoint, the answer to the counseling sheets was more complete and helpful.

Oral presentation is an essential skill for clinical nutritionists that should be seriously considered in the educational curriculum, as it enhances students' skills and learning as well as the interaction and collaboration of the medical team.

## Data Availability Statement

The raw data supporting the conclusions of this article will be made available by the authors, without undue reservation.

## Author Contributions

All authors reviewed and commented on subsequent drafts of the manuscript.

## Conflict of Interest

The authors declare that the research was conducted in the absence of any commercial or financial relationships that could be construed as a potential conflict of interest.

## Publisher's Note

All claims expressed in this article are solely those of the authors and do not necessarily represent those of their affiliated organizations, or those of the publisher, the editors and the reviewers. Any product that may be evaluated in this article, or claim that may be made by its manufacturer, is not guaranteed or endorsed by the publisher.
