# Erratum: Implementation of a Case Presentation Program for Clinical Nutrition Students

**DOI:** 10.3389/fnut.2022.937569

**Published:** 2022-05-31

**Authors:** Frontiers Production Office

**Affiliations:** Frontiers Media SA, Lausanne, Switzerland

**Keywords:** case-based learning, questionnaire, student, clinical nutrition, consultation

Due to a production error, Saba Vahdatshariatpanahi was not included as an author in the published article.

The publisher apologizes for this mistake. The original version of this article has been updated.

